# Marked and rapid effects of pharmacological HIF-2**α** antagonism on hypoxic ventilatory control

**DOI:** 10.1172/JCI133194

**Published:** 2020-03-23

**Authors:** Xiaotong Cheng, Maria Prange-Barczynska, James W. Fielding, Minghao Zhang, Alana L. Burrell, Joanna D.C.C. Lima, Luise Eckardt, Isobel L.A. Argles, Christopher W. Pugh, Keith J. Buckler, Peter A. Robbins, Emma J. Hodson, Richard K. Bruick, Lucy M. Collinson, Fraydoon Rastinejad, Tammie Bishop, Peter J. Ratcliffe

**Affiliations:** 1 Target Discovery Institute and; 2 Ludwig Institute for Cancer Research, University of Oxford, Oxford, United Kingdom.; 3 Francis Crick Institute, London, United Kingdom.; 4 Department of Physiology, Anatomy and Genetics, University of Oxford, Oxford, United Kingdom.; 5 Department of Biochemistry, University of Texas Southwestern Medical Center, Dallas, Texas, USA.

**Keywords:** Therapeutics, Cancer, Respiration, hypoxia

## Abstract

Hypoxia-inducible factor (HIF) is strikingly upregulated in many types of cancer, and there is great interest in applying inhibitors of HIF as anticancer therapeutics. The most advanced of these are small molecules that target the HIF-2 isoform through binding the PAS-B domain of HIF-2α. These molecules are undergoing clinical trials with promising results in renal and other cancers where HIF-2 is considered to be driving growth. Nevertheless, a central question remains as to whether such inhibitors affect physiological responses to hypoxia at relevant doses. Here, we show that pharmacological HIF-2α inhibition with PT2385, at doses similar to those reported to inhibit tumor growth, rapidly impaired ventilatory responses to hypoxia, abrogating both ventilatory acclimatization and carotid body cell proliferative responses to sustained hypoxia. Mice carrying a HIF-2α PAS-B S305M mutation that disrupts PT2385 binding, but not dimerization with HIF-1β, did not respond to PT2385, indicating that these effects are on-target. Furthermore, the finding of a hypomorphic ventilatory phenotype in untreated HIF-2α S305M mutant mice suggests a function for the HIF-2α PAS-B domain beyond heterodimerization with HIF-1β. Although PT2385 was well tolerated, the findings indicate the need for caution in patients who are dependent on hypoxic ventilatory drive.

## Introduction

Hypoxia-inducible factors (HIFs) are transcriptional factors that mediate a wide range of adaptive responses to low oxygen and play a central role in the maintenance of oxygen homeostasis (reviewed in refs. 1–3). As disturbances of oxygen homeostasis are critical to many aspects of human disease, there is great interest in developing agents that modulate HIF pathways and in defining the benefits and risks of their therapeutic use (4, 5).

The HIF DNA binding complex is composed of an α/β heterodimer of basic helix-loop-helix (bHLH) PAS proteins. Oxygen-dependent activity of HIF is conveyed by the HIF-α subunit; in human cells, there are 3 HIF-α isoforms, of which HIF-1α and HIF-2α are the most strongly expressed and best understood. In cancer, HIFs are frequently upregulated either by microenvironmental hypoxia within the tumor or by genetic connections to mutant oncogene or tumor-suppressor pathways (reviewed in refs. 1–3). Thus, HIF constitutes a key “growth support” pathway for cancer, enhancing oxygen delivery through angiogenesis and promoting a range of tumor-associated phenotypes. In some cancers, it has further been proposed that the activation of HIF not only supports tumor growth, but drives its progression. One example is clear cell renal cell carcinoma (ccRCC), the most common form of human kidney cancer (6, 7). These tumors frequently manifest biallelic inactivation of the von Hippel–Lindau tumor-suppressor pVHL, a ubiquitin E3 ligase that normally targets HIF-α for destruction in the presence of oxygen (reviewed in refs. 6, 7). Inactivation of pVHL leads to constitutive stabilization of HIF-α in ccRCC. Interestingly, ccRCC commonly manifests a strong bias toward expression of HIF-2α, and genetic interventions on HIF-2α in ccRCC cells grown as tumor xenografts suggest that this isoform is necessary and sufficient to drive ccRCC (8, 9). These findings have led to great interest in the development of therapeutic agents that antagonize HIFs, either specifically or generally, for the treatment of cancer.

The most advanced of these approaches is represented by molecules that specifically target a pocket in the PAS-B domain of HIF-2α to allosterically destabilize the heterodimeric complex involving HIF-2α with HIF-1β and hence inhibit HIF-2 transcriptional activity (10, 11). These antagonists have shown efficacy in preclinical models using ccRCC cell lines or patient-derived ccRCC xenografts (12–14) and in early phase clinical trials on therapy-resistant advanced ccRCC (15, 16). These molecules are also undergoing clinical trials in other cancers where the HIF-2α isoform is considered to be particularly important (17) and in ccRCC, which is associated with inherited von Hippel–Lindau disease (18).

However, because HIF, including HIF-2, is involved in many aspects of normal physiology, a key question arises as to whether such agents affect the function of nonneoplastic tissues and, if so, at what dose and under which circumstances. One important response in which HIF, in particular HIF-2, has been implicated by genetic interventions is in the control of respiration by the carotid body (19–21). However, in common with most studies that deploy genetic inactivation technology, such studies of HIF genetic inactivation generally aim to generate complete loss of a specific HIF-α protein in a specific cell type and do not necessarily mimic dose-dependent actions of a pharmaceutical agent that is applied systemically.

As interventions on the HIF system move forward through clinical trials, there is a pressing need to understand better their effects on systemic physiology. To that end, we have examined the effects of PT2385, one of the HIF-2α antagonists that shows clinical efficacy in ccRCC (15), on morphological changes and hypoxic ventilatory control that are mediated by the carotid body. We report that PT2385 blocks the proliferative and ultrastructural changes in the cells within this organ that are observed in response to hypoxia. Enhanced ventilatory sensitivity to hypoxia was rapidly and severely ablated at the doses of PT2385 for which antitumor effects have been reported (14). These effects were not observed in recombinant mice containing a mutation in *Epas1* (the gene encoding HIF-2α), which largely ablates the binding of PT2385 to HIF-2α, indicating that they represent on-target effects of the molecule.

## Results

### Effects of PT2385 on ventilatory sensitivity to hypoxia.

In response to breathing a hypoxic atmosphere, pulmonary ventilation increases rapidly within seconds, then more progressively over hours to days as hypoxia is sustained, a phenomenon that is termed ventilatory acclimatization (22). These responses involve a progressive increase in ventilatory sensitivity to chemoreceptive stimuli in the carotid body. Because in clinical or physiological settings, such as chronic lung disease or altitude exposure, hypoxia is generally sustained and because genetic studies have suggested an important role for HIF-2 in this setting (19, 20, 23, 24), we first studied the effects of PT2385 on ventilatory sensitivity in animals exposed to sustained hypoxia.

**Figure 1:**
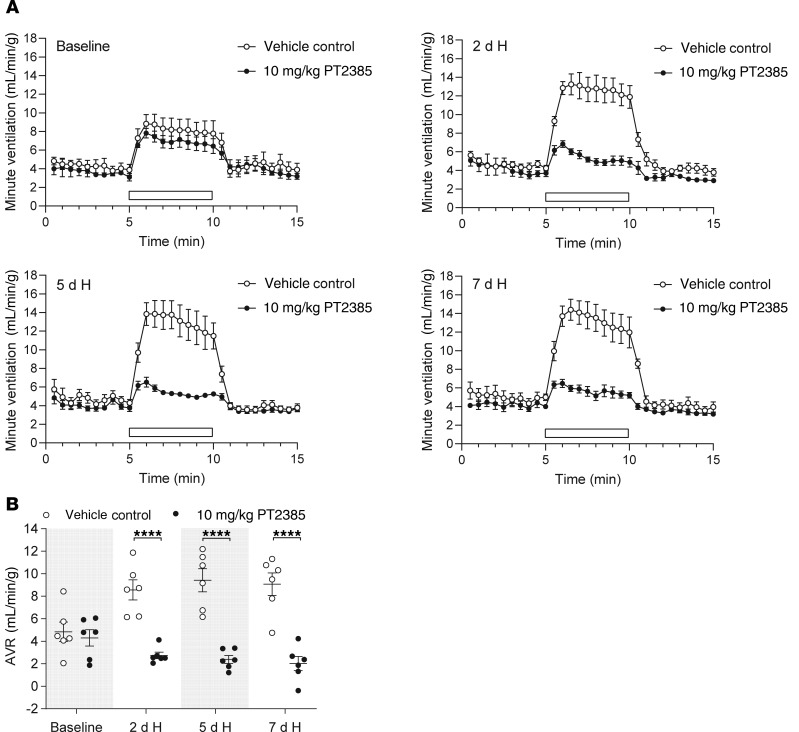
PT2385 ablates ventilatory acclimatization to hypoxia in WT male mice. (**A**) Graphs show changes in minute ventilation in response to an acute (5 minutes) challenge with 10% O_2_/3% CO_2_ (white bars) in male mice before (baseline) and following twice daily treatment with 10 mg/kg PT2385 (or vehicle), beginning 24 hours before 7-day exposure to hypoxia (H, 10% oxygen) and continuing throughout (to a total of 8 days of treatment) (*n* = 6). (**B**) Graph shows AVRs to challenges with 10% O_2_/3% CO_2_, quantified from the minute ventilation shown in **A**. Data were analyzed by 2-way repeated measures ANOVA with baseline recordings removed from the statistical analysis (*P* values in Table 1), followed by Holm-Sidak’s multiple comparisons 2-tailed test for individual time point comparisons, for which significance is reported in the graph. ****P < 0.0001.

Male mice were treated by gavage with 3 or 10 mg/kg PT2385 (or vehicle alone) twice daily, doses that have been shown to be effective in reducing tumor size in ccRCC xenografts in mice (14). PT2385 was commenced 24 hours before and maintained throughout the 7-day exposure of mice to hypoxia. To monitor changes in ventilatory sensitivity during this period, mice were removed from the normobaric altitude chamber, placed into the plethysmograph, and allowed to equilibrate to normal air, then subjected to defined acute (5 minutes) gas challenges (10% oxygen alone, 10% oxygen with 3% carbon dioxide, and 3% carbon dioxide alone) before the start of the experiment and after 2, 5, and 7 days of sustained hypoxia. Acute (5 minutes) challenge with 10% oxygen alone elicits a poorly sustained increase in ventilation due to hyperventilation-induced hypocapnia. Therefore, the challenge of 10% oxygen with 3% carbon dioxide (calculated to provide approximate compensation for hypocapnia) was used as the main test gas to approximate isocapnic hypoxia, as described previously (19, 20, 25). Ventilatory responses to all 3 gas challenges are shown in Figure 1, Table 1, and Supplemental Figures 1 and 2 (supplemental material available online with this article; https://doi.org/10.1172/JCI133194DS1).

Progressive increases in ventilatory sensitivity were observed with sustained hypoxia in control mice treated with vehicle alone, with marked increases in responses to gas challenges after 2 days at 10% oxygen that were further enhanced after 5 and 7 days at 10% oxygen (Figure 1, Supplemental Figure 1, and Table 1). Strikingly, treatment with PT2385 at both 3 and 10 mg/kg doses essentially ablated these increases in ventilatory sensitivity (Figure 1, Supplemental Figure 1, and Table 1). For example, acute ventilatory responses (AVRs) to 10% oxygen with 3% carbon dioxide after 7 days of hypoxia were reduced from 9.22 ± 1.60 mL/min/g in vehicle-treated mice to 4.24 ± 0.90 mL/min/g in mice treated with 3 mg/kg PT2385 and were similar to those at baseline, i.e., before sustained hypoxia (4.18 ± 0.43 mL/min/g). In the case of the 10 mg/kg dose, AVRs of mice treated with PT2385 and in 7-day hypoxia were lower than those at baseline (2.02 ± 0.62 versus 4.31 ± 0.73 mL/min/g). Similar observations were made when 10% oxygen was used as the acute gas challenge, although, as discussed above, these responses were less sustained (Table 1 and Supplemental Figure 2). Interestingly, PT2385 treatment also reduced responses to 3% carbon dioxide in this setting (Table 1). In humans, ventilatory acclimatization to hypoxia is known to increase the sensitivity of the central chemoreflex response to carbon dioxide, so as to increase the slope of the relationship between minute ventilation and end-tidal carbon dioxide (26). Hence, the reduced sensitivity of PT2385-treated mice to carbon dioxide in this setting is also consistent with an action of PT2385 on ventilatory acclimatization to hypoxia. In summary, these findings reveal that treatment with PT2385 exerts striking effects on ventilatory control, essentially ablating the enhanced ventilatory sensitivity, or ventilatory acclimatization that occurs during sustained exposure to hypoxia. We further sought to determine whether PT2385 has similar effects on hypoxic ventilatory control in female mice compared with those observed in male mice. As with male mice, ventilatory acclimatization was abolished in female mice treated with either 3 or 10 mg/kg PT2385 (Supplemental Figure 3 and Supplemental Table 1).

Interestingly, neither 3 nor 10 mg/kg PT2385 treatment during sustained hypoxia significantly altered hematocrits in male or female mice compared with vehicle-treated control mice in hypoxia (Supplemental Figure 4). This may be because the failure to acclimatize with PT2385 treatment results in greater hypoxemia and a higher hypoxic drive on erythropoietin expression; indeed, 10 mg/kg PT2385 treatment over the equivalent time course (8 days) in normoxic male mice does result in a significant reduction in hematocrits (Supplemental Figure 4), in line with the anemia observed in patients treated with PT2385 (15).

**Figure 2:**
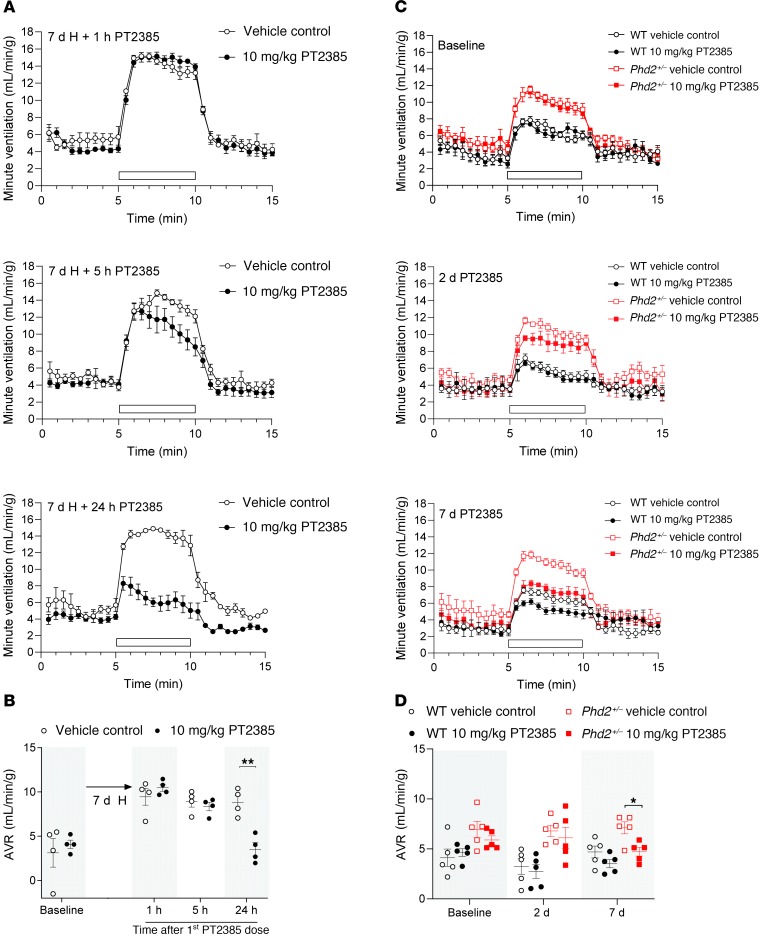
Reversal of enhanced ventilatory sensitivity in acclimatized WT and *Phd2^+/–^* male mice by PT2385. Graphs illustrate changes in minute ventilation in response to an acute challenge with 10% O_2_/3% CO_2_ (white bars) before (baseline) and at the indicated times after the onset of twice daily treatment with 10 mg/kg PT2385. (**A**) Male mice were preexposed to 7 days of hypoxia (H, 10% oxygen), then treated with PT2385 (or vehicle) in continuing hypoxia (*n* = 4). (**C**) *Phd2^+/–^* or littermate WT male mice were treated with PT2385 (or vehicle) and maintained in normal air (*n* = 5). (**B** and **D**) Summary of AVRs calculated from data shown in **A** and **C** and from measurements taken before the start of the experiment in **A** (baseline). Data from **B** and **D** were analyzed by Holm-Sidak’s multiple comparisons 2-tailed tests (for all time points within each genotype for **D**), for which significance is reported in the graphs. *P < 0.05; **P < 0.01.

To pursue the effects on ventilatory acclimatization further, we next tested to determine whether PT2385 treatment could reverse enhanced ventilatory sensitivity that has already been established, either through prior exposure to sustained hypoxia or in the setting of pseudohypoxia following genetic inactivation of a negative regulator of HIF. To test the former, WT male mice were first exposed to 10% oxygen for 7 days, after which they were treated with 10 mg/kg PT2385 or vehicle twice daily and maintained in hypoxia (Figure 2, A and B). Ventilatory sensitivity was measured using the same acute 5-minute challenges with test gases, as described above. PT2385 treatment progressively reversed enhanced ventilatory sensitivity, reducing the responses to those observed in unacclimatized mice within 24 hours (Figure 2, A and B). To test the effects of PT2385 in the setting of pseudohypoxic enhancement of ventilatory sensitivity, male mice with heterozygous inactivation of HIF prolyl hydroxylase 2 (*Phd2*, the principal PHD isoform which negatively regulates HIF; refs. 27, 28) were treated with 10 mg/kg PT2385. As reported (20, 25), *Phd2^+/–^* mice displayed enhanced hypoxic ventilatory sensitivity compared with their WT littermates (Figure 2, C and D). These responses were also reduced by PT2385, although interestingly, this appeared to require longer exposure to the compound. Whereas PT2385 completely reversed enhanced ventilatory sensitivity to environmental hypoxia within 24 hours in WT mice (Figure 2, A and B), the enhanced sensitivity in *Phd2^+/–^* mice was progressively reduced after between 2 and 7 days of treatment with PT2385 (Figure 2, C and D). The delayed onset of the drug effect in *Phd2^+/–^* (versus acclimatized WT) mice likely reflects the constitutive nature of the genetic intervention, whose long-term consequences may be slow to reverse. Indeed, *Phd2^+/–^* mice have enlarged carotid bodies that may contribute to their exaggerated hypoxic ventilatory responses (25). Together, these results demonstrate that, as well as preventing its development, PT2385 can reverse ventilatory acclimatization to hypoxia and pseudohypoxia.

**Figure 3:**
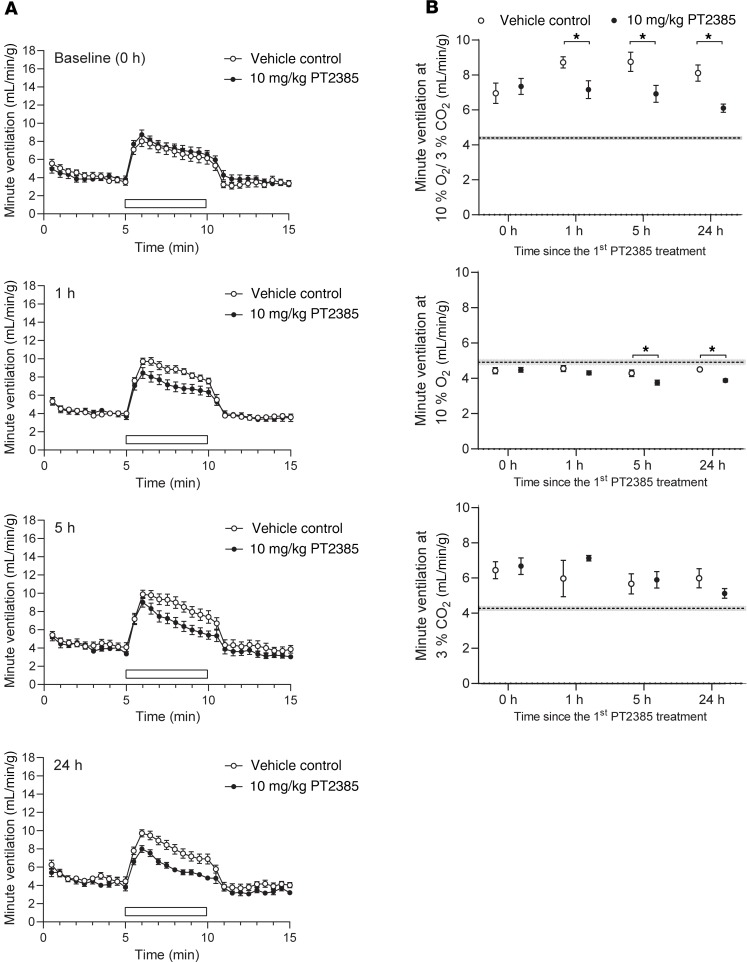
Rapid reduction in ventilatory sensitivity to hypoxia in unacclimatized PT2385-treated male mice. (**A**) Graphs show changes in minute ventilation in response to an acute (5 minutes) challenge with 10% O_2_/3% CO_2_ (white bars). (**B**) Graphs show average minute ventilation during 5-minute challenges with 10% O_2_/3% CO_2_ (upper panel), 10% O_2_ (middle panel), or 3% CO_2_ (lower panel). Measurements for **A** and **B** were made before (baseline, 0 hours) and 1, 5, and 24 hours after the first 10 mg/kg PT2385 (or vehicle) dose (*n* = 15, except for 3% CO_2_ at 1 hour where *n* = 3); second dose of PT2385 was given immediately after the 5-hour measurements. Data were analyzed by Holm-Sidak’s multiple comparisons 2-tailed tests, for which significance is reported in the graphs. *P < 0.05. Dotted lines show the average resting minute ventilation in air before the acute gas challenge, across all time points and treatment groups. Data are shown as mean ± SEM (shaded area around the dotted line).

Interestingly, in addition to reducing the enhanced ventilatory sensitivity in *Phd2^+/–^* mice, we noted that PT2385 appeared to reduce ventilatory sensitivity even in their WT littermates (Figure 2, C and D). Such an effect on baseline (unacclimatized) ventilatory sensitivity was also suggested by a reduction in sensitivity below the baseline in mice exposed to 7 days of hypoxia and treated with PT2385 at a dose of 10 mg/kg (Figure 1 and Table 1). Therefore, we also performed experiments to assess the effects of PT2385 on baseline ventilatory sensitivity, including the dose and time dependence of any activity. In these experiments, WT male mice were treated with PT2385 or vehicle and their ventilatory sensitivity measured after 1, 5, and 24 hours, using the same acute (5 minutes) gas challenges as described above. Since baseline AVRs in unacclimatized mice are small relative to confounding behavioral variations in ventilation when test gases are introduced to the plethysmograph, quantitative data are presented as the average ventilation over the entire period of acute gas exposure (Figure 3B, Supplemental Figure 5A, and Supplemental Figure 6). Responses are shown in full for selected experiments in Figure 3A. These experiments revealed a clear, though partial, loss of sensitivity to gas challenge with 10% oxygen with 3% carbon dioxide in mice treated with PT2385 at 10 mg/kg (Figure 3). This reduction in ventilatory sensitivity was similar at all time points, including 1 hour after the first dose of PT2385, and was most clearly manifest as a reduction in tidal volume as opposed to respiratory rate (Supplemental Figure 5A). Interestingly, these effects were not observed with PT2385 at 3 mg/kg (Supplemental Figure 6), suggesting different dose dependence from actions of PT2385 in acclimatized mice (Figure 1, Supplemental Figure 1, and Table 1). Of significance, there were no differences in ventilatory sensitivity to carbon dioxide at either dose (Figure 3B and Supplemental Figure 6), suggesting that PT2385 primarily alters hypoxic, but not hypercapnic, responses. Similar effects on acute hypoxic ventilatory control were observed when WT female mice were treated with 3 or 10 mg/kg (Supplemental Figure 5B and Supplemental Figure 7). Together, these data suggest that PT2385, in addition to ablating ventilatory acclimatization, exerts rapid effects (within 1 hour) on acute hypoxic ventilatory control.

### PT2385 treatment prevents carotid body cellular and proliferative responses to sustained hypoxia.

**Figure 4:**
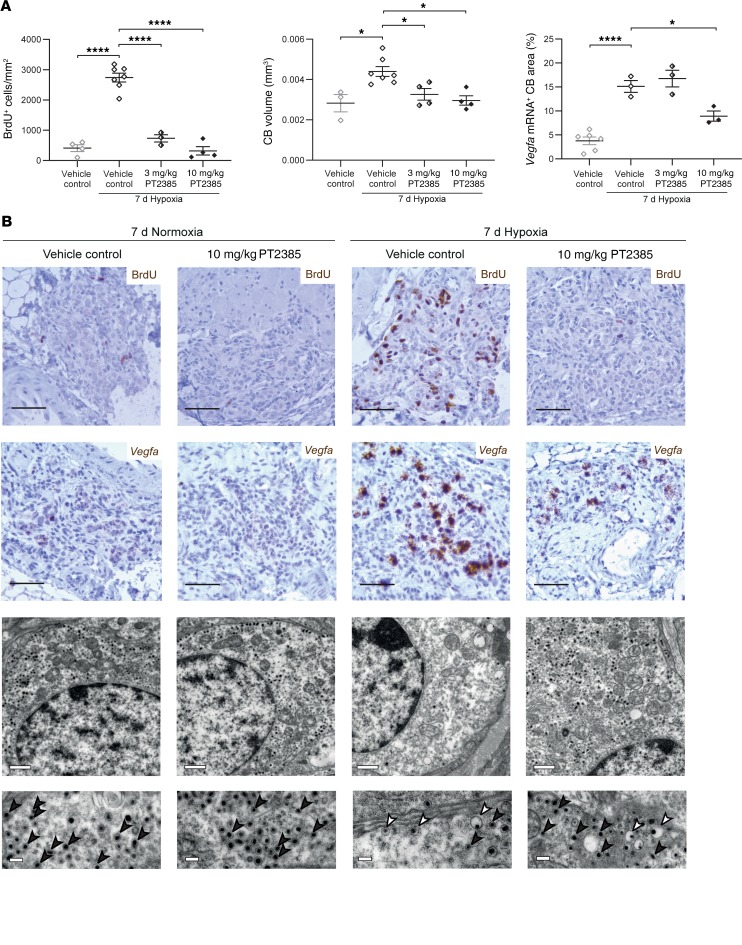
Effects of PT2385 on cellular responses to hypoxia in the carotid body. (**A**) Morphometry and (**B**) representative images of carotid bodies (CBs) from WT male mice exposed to 7 days of hypoxia (10% oxygen) or normoxia and treated twice daily with 3 or 10 mg/kg PT2385 (or vehicle), beginning 24 hours before and continuing throughout 7-day exposure to hypoxia (10% oxygen) or normoxia (to a total of 8 days of treatment). (**A**) Quantification of BrdU^+^ cells per mm^2^ (left panel), CB volumes (middle panel), and *Vegfa* mRNA^+^ CB area (right panel). Data were analyzed by 1-way ANOVA, followed by Holm-Sidak’s multiple comparisons 2-tailed tests, for which significance is reported in the graphs. *P < 0.05; ****P < 0.0001. (**B**) Immunostaining for BrdU; *Vegfa* mRNA in situ hybridization; representative electron micrographs showing the DCVs in type I cells of the CB (note that changes induced by hypoxia are not observed in mice treated with PT2385; compare third and fourth columns) (*n* = 2). Scale bars: 50 μm (top 2 panels); 1 μm (top electron micrograph panels); and 200 nm (bottom electron micrograph panels). Black arrowheads show typical DCVs, while white arrowheads show “eccentric” DCVs. Images of carotid bodies of mice treated with 3 mg/kg PT2385 are shown in Supplemental Figure 8.

Along with ventilatory changes, sustained hypoxia induces proliferation of multiple cell types within the carotid body, resulting in hyperplasia of the organ (19, 29). To analyze the effects of PT2385 on this cellular proliferative response, BrdU incorporation was measured in male mice treated with PT2385 or vehicle and subjected to the same sustained exposure to hypoxia as used in the ventilatory experiments (7 days at 10% oxygen) or maintained in normal air. In animals given BrdU and maintained for 7 days in normal air, few BrdU-positive cells were observed in carotid bodies from either PT2385- or vehicle-treated mice. Following 7 days at 10% oxygen, striking incorporation of BrdU across multiple cell types (including type I and endothelial cells) in the carotid body (but not in surrounding tissues such as the supracervical ganglion) was observed in vehicle-treated mice (Figure 4 and data not shown). Treatment of the hypoxia-exposed mice with either 3 or 10 mg/kg PT2385 twice daily resulted in near-total loss of BrdU-positive cells across all cell types (Figure 4 and Supplemental Figure 8) and prevented the associated increase in carotid body size (Figure 4A). Although the circuitry mediating proliferative responses of the carotid body to hypoxia are not well understood, the HIF transcriptional target gene *Vegfa* has been reported to be induced by hypoxia in type I cells (19) and may be important in mediating proliferative responses by paracrine signaling to other neighboring cell populations. Responses of *Vegfa* mRNA to hypoxic exposure were therefore also measured in these animals by in situ hybridization. The marked induction of *Vegfa* mRNA observed in type I cells of hypoxic carotid bodies was also greatly reduced by 10, but not 3, mg/kg PT2385 treatments (Figure 4 and Supplemental Figure 8). Female mice subjected to the same sustained hypoxia protocol as above exhibited high levels of proliferation and *Vegfa* mRNA expression in the carotid body, as observed in male mice; these hypoxia-induced changes were likewise prevented by concomitant twice daily 10, and to a lesser extent 3, mg/kg PT2385 treatments (Supplemental Figure 9).

In addition to proliferative changes in type I cells, sustained hypoxia induces HIF-2α–dependent changes to type I cell ultrastructure, as assessed by electron microscopy (EM) (19). These hypoxia-induced changes include a reduction in dense core vesicles (DCVs) in type I cells and an increased frequency of “eccentric” DCVs with an expanded vesicle and a displaced dense core (19, 30). Strikingly, PT2385 treatment of hypoxic male mice prevented these changes, such that DCVs appeared similar to those observed in mice breathing normal air (Figure 4B), indicating that HIF-2α has a role, directly or indirectly, in the secretory process itself, for example, by altering the expression of gene(s) involved in the regulation of secretion and/or DCV content. Together, these data suggest that PT2385 blocks both proliferation and oxygen-dependent vesicular functions of type I cells of the carotid body and that both these changes might contribute to reduced ventilatory sensitivity and an inability of PT2385-treated mice to acclimatize to hypoxia.

**Figure 5:**
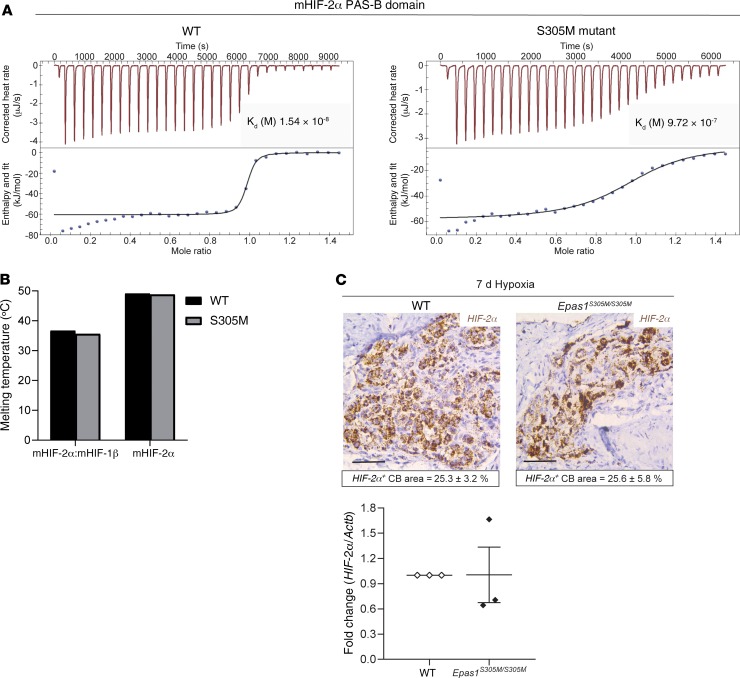
The S305M mutation prevents binding of PT2385 to the HIF-2α PAS-B domain without disrupting HIF-2α stability or expression. (**A**) ITC of WT and S305M mutant mouse HIF-2α PAS-B domain with PT2385, demonstrating the effect of the mutation on the PT2385 dissociation constant (K_d_). (**B**) Thermal shift assay; no effects of S305M mutation are observed on the stability of mouse HIF-2α alone or in complex with HIF-1β. (**C**) Expression of *HIF-2α* mRNA in carotid bodies of WT and *Epas1^S305M/S305M^* mice exposed to 7 days of hypoxia (10% oxygen), as assessed by in situ hybridization (Scale bars: 50 μm. Quantified *HIF-2α* mRNA^+^ carotid body area is depicted in boxes below the relevant images, *n* = 4) or quantitative real-time PCR. Values are the mean fold change of *Hif-2α* mRNA relative to *Actb* mRNA, from 3 experiments on CBs pooled from 5 animals per genotype in each; data analyzed by a 2-tailed *t* test, *P* > 0.05.

*The S305M mutation in HIF-2αconfers resistance to PT2385 without altering HIF-2α protein stability or mRNA expression*. The class of HIF-2α antagonists that includes PT2385 binds directly to a pocket within the PAS-B domain of the HIF-2α, but not HIF-1α, protein and allosterically weakens HIF-2α/HIF-1β heterodimer stability (10, 11, 31). To investigate the specificity of this action, the effect of PT2385 on HIF-2α bearing a point mutation located in the HIF-2α PAS-B domain (S305M in the mouse sequence; ref. 32) was tested. This mutation was originally selected based on its location and ability to interfere with the binding of other HIF-2α antagonists acting within the same binding site (10). The mutant methionine residue could in principle block the drug-binding pocket locally or alter the overall conformation and stability of the protein to weaken drug binding. Using proteins expressed and purified in *E*. *coli* and isothermal titration calorimetry (ITC), we measured the equilibrium dissociation constant (K_d_) of PT2385 binding to WT mouse HIF-2α PAS-B domain to be 15.4 nM. By comparison, the K_d_ of PT2385 binding to mouse HIF-2α S305M mutant PAS-B domain was 0.97 μM, indicating a more than 60-fold loss in binding affinity (Figure 5A). These biochemical measurements confirmed that PT2385 binding is substantially reduced in the context of this point mutation. We then investigated whether loss of PT2385 binding was due to binding-site occlusion or a loss of overall protein stability caused by the point mutation. Protein thermal shift assays were applied to determine the melting temperature (T_m_) of S305M and WT mouse HIF-2α purified proteins. T_m_ values were similar for WT and S305M HIF-2α proteins, in the context of both their isolated PAS-B domains alone and their HIF-2α/HIF-1β heterodimers consisting of their larger bHLH-PAS-A-PAS-B segments (Figure 5B). Closely similar T_m_ values for WT and S305M proteins indicate that the point mutation does not alter the global fold or stability of HIF-2α protein or the stability of its heterodimeric complex with HIF-1β. We infer that the loss of PT2385 binding to the S305M protein is due to the larger methionine (versus serine) side chain locally occluding the PAS-B pocket where PT2385 physically binds, causing a more than 60-fold loss of binding affinity.

Mice engineered to carry this mutation constitutively (herein referred to as *Epas1^S305M/S305M^* mice) have been described as having broadly unaffected levels of *Hif-2α* mRNA and protein expression in the kidney (33). To determine whether this is the case in the carotid body, *Hif-2α* mRNA levels were assessed by in situ hybridization and quantitative real-time PCR in carotid bodies from *Epas1^S305M/S305M^* mice (Figure 5C). *Hif-2α* mRNA was abundantly expressed in carotid bodies from WT mice, and levels were similarly high in carotid bodies from *Epas1^S305M/S305M^* mice (Figure 5C). Taken together, these findings indicate that the S305M mutation confers resistance to PT2385 without altering the expression or conformation of HIF-2α.

**Figure 6:**
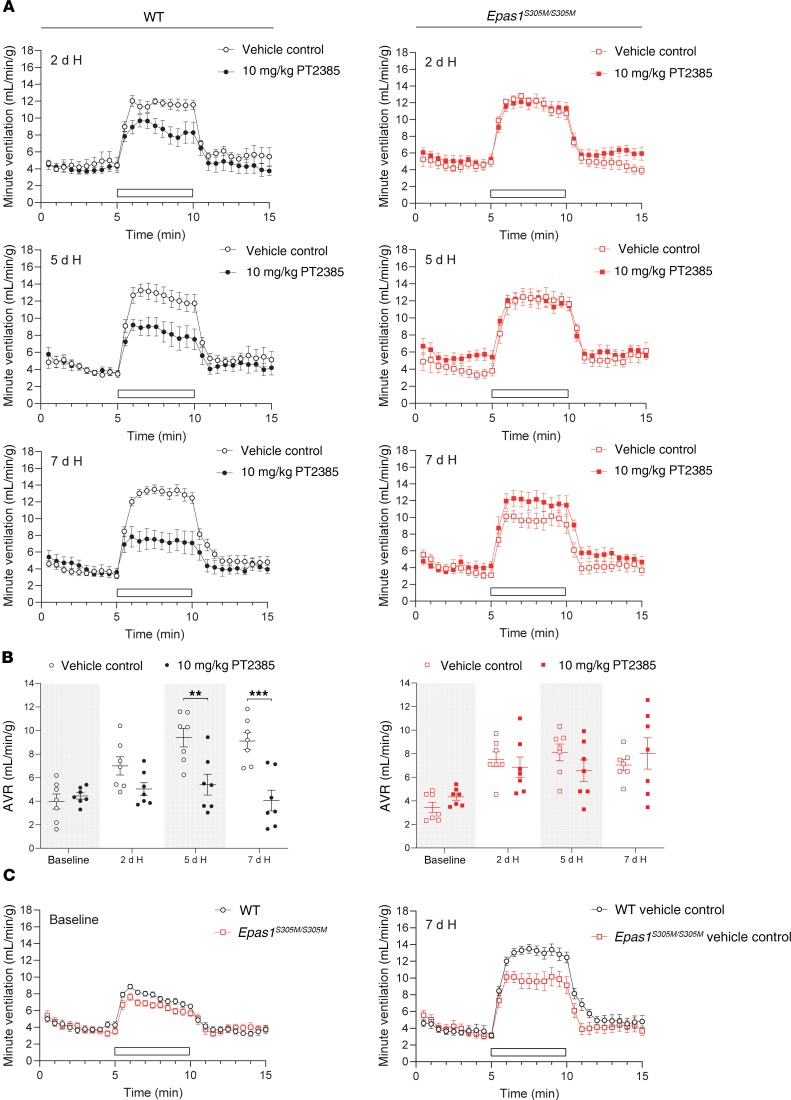
*Epas1^S305M/S305M^* male mice are resistant to PT2385 effects on enhanced ventilatory sensitivity to hypoxia. Graphs show minute ventilation in response to an acute (5 minutes) challenge with 10% O_2_/3% CO_2_ (white bars) before (baseline) and after the indicated exposure to hypoxia (H, 10% oxygen). (**A**) Comparison of WT and *Epas1^S305M/S305M^* male mice treated twice daily with 10 mg/kg PT2385 (or vehicle), beginning 24 hours before 7 days of exposure to hypoxia and continuing throughout (to a total of 8 days of treatment) (*n* = 7). (**B**) Graphs show AVRs to challenges with 10% O_2_/3% CO_2_, quantified from the minute ventilation in **A**. Data from each genotype were analyzed by 2-way repeated measures ANOVA, with baseline recordings removed from statistical analyses (*P* values in Table 2), followed by Holm-Sidak’s multiple comparisons 2-tailed tests for individual time point comparisons, for which significance is reported in the graphs. **P < 0.01; ***P < 0.001. (**C**) Comparison of ventilatory sensitivity in vehicle-treated WT and *Epas1^S305M/S305M^* male mice before (baseline) and after 7 days of exposure to hypoxia. For baseline comparison, measurements were pooled from all treatment groups per genotype (*n* = 14). Data at 7 days are replotted from **A** to provide direct comparison (*n* = 7).

*PT2385 has no effect on ventilatory or carotid body responses to hypoxia in Epas1^S305M/S305M^mice*. To test whether ventilatory sensitivity is altered by PT2385 in *Epas1^S305M/S305M^* mice, male *Epas1^S305M/S305M^* mice and WT littermate controls were exposed to hypoxia and administered 10 mg/kg PT2385 or vehicle twice daily. As before, ventilatory sensitivity was assessed before the start of PT2385 treatment and after exposure to periods of sustained hypoxia of up to 7 days (Figure 6 and Table 2). In striking contrast to WT mice, treatment of *Epas1^S305M/S305M^* mice with PT2385 did not reduce ventilatory sensitivity at any time point during sustained hypoxia (Figure 6, A and B, and Table 2). Interestingly, although manifesting clear increases in ventilatory sensitivity after 2 days of hypoxia, untreated *Epas1^S305M/S305M^* mice did not show further increases as hypoxia was maintained, their ventilatory sensitivity after 7 days exposure to hypoxia being clearly reduced compared with that of WT littermates (7.04 ± 0.47 versus 9.11 ± 0.72 mL/min/g, Student’s 2-tailed *t* test *P* = 0.032) (Figure 6C and Table 2).

**Figure 7:**
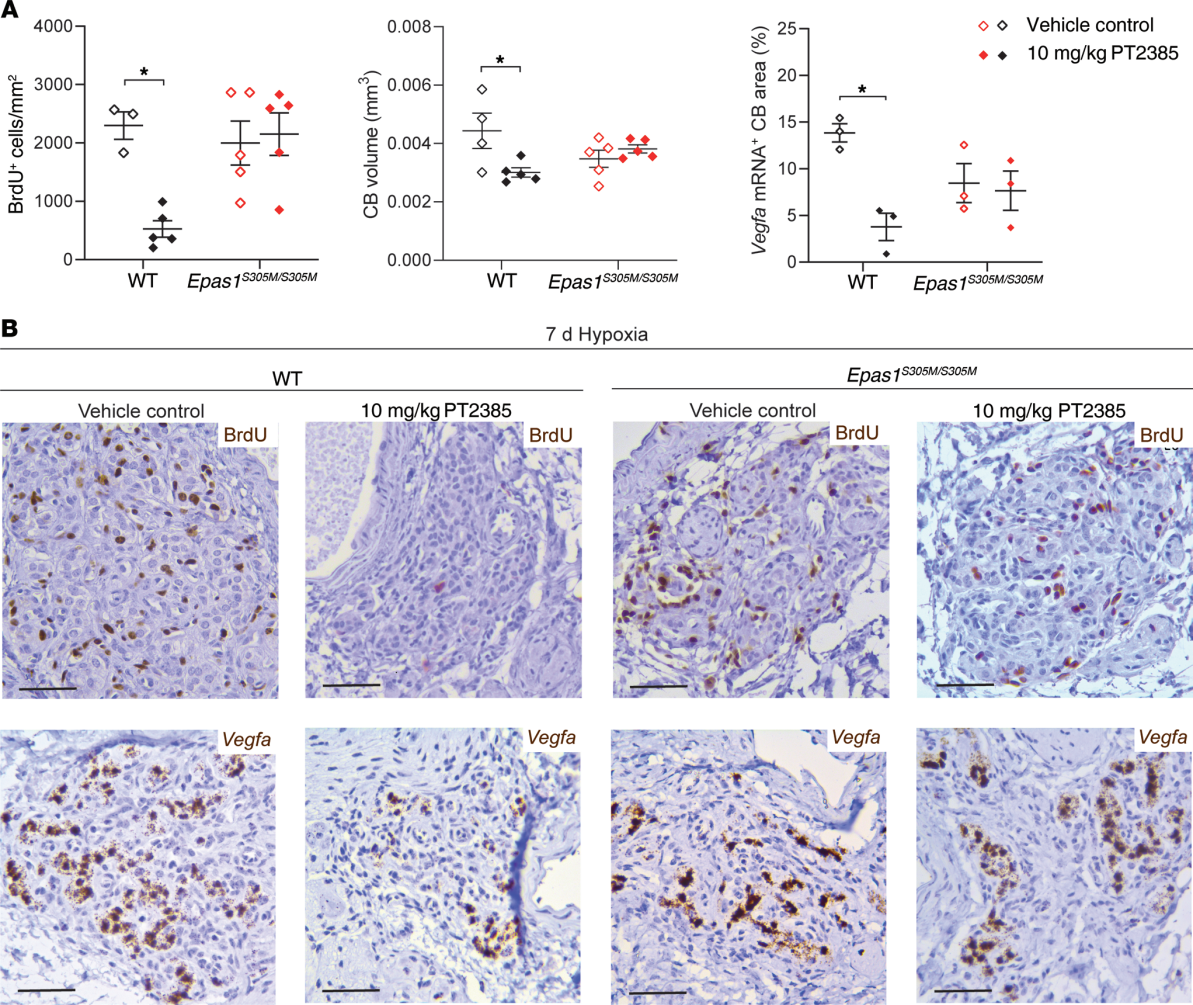
Effects of PT2385 on cellular responses to hypoxia in carotid bodies of *Epas1^S305M/S305M^* male mice. (**A**) Morphometry and (**B**) representative images of carotid bodies from WT and *Epas1^S305M/S305M^* mice treated twice daily with 10 mg/kg PT2385 (or vehicle) beginning 24 hours before and continuing throughout 7 days of exposure to hypoxia (10% oxygen) (to a total of 8 days of treatment). (**A**) Quantification of BrdU^+^ cells per mm^2^ (left panel), CB volumes (middle panel), *Vegfa* mRNA^+^ CB area (right panel). Data were analyzed by 2-way ANOVA followed by Holm-Sidak’s multiple comparisons 2-tailed tests, for which significance is reported in the graphs. *P < 0.05. (**B**) Immunostaining for BrdU and in situ hybridization for *Vegfa* mRNA. Scale bars: 50 μm.

To assess whether *Epas1^S305M/S305M^* mice are also resistant to PT2385-mediated effects on the cellular and proliferative responses to hypoxia, carotid bodies were examined from the same *Epas1^S305M/S305M^* mice and compared with the same littermate control mice as were used for the ventilatory measurements described above. High levels of BrdU incorporation were observed in WT mice following 7 days at 10% oxygen and were largely abolished by twice daily treatment with 10 mg/kg PT2385 (Figure 7). Although there was greater variability in this response, with regional variations in BrdU incorporation, this response (and the accompanying carotid body hyperplasia) was unaffected by PT2385 treatment in *Epas1^S305M/S305M^* littermate mice (Figure 7). As with earlier experiments (Figure 4), *Vegfa* mRNA was strongly induced in type I cells of the carotid body by exposure of the WT mice to 10% oxygen for 7 days; this induction of *Vegfa* mRNA was largely ablated by PT2385 (Figure 7). In contrast, PT2385 had little or no effect on hypoxia-induced *Vegfa* mRNA levels in type I cells in littermate mice of the *Epas1^S305M/S305M^* genotype (Figure 7).

Taken together, these findings indicate that *Epas1^S305M/S305M^* mice are resistant to the effects of PT2385 on ventilatory sensitivity and cellular changes in the carotid body that are induced by sustained hypoxia, consistent with these actions of PT2385 being on-target to HIF-2α. Unexpectedly, responses to sustained hypoxia were also reduced in untreated *Epas1^S305M/S305M^* mice when compared with WT littermates.

## Discussion

Our findings reveal major effects of the small molecule HIF-2α antagonist PT2385 on hypoxic ventilatory control. Particularly striking were effects on the enhanced ventilatory sensitivity that develops after exposure to a period of sustained hypoxia. This response was essentially ablated at PT2385 doses that are identical to those showing efficacy in studies of patient-derived ccRCC xenografts in the mouse (14). Hypoxic ventilatory sensitivity is considered to be largely mediated by the carotid body, which also responds to sustained hypoxia with changes in gene expression, ultrastructural alterations to DCVs in the secretory type I cells, and a marked multilineage cellular proliferation (19, 34). All these responses were abrogated, or greatly reduced, by PT2385. In contrast, such effects were not seen, or were markedly reduced, in mice bearing a mutation in the HIF-2α PAS-B pocket, which was shown to greatly reduce binding of PT2385 to the HIF-2α polypeptide, strongly suggesting that they represent specific on-target effects exerted through an action on HIF-2α.

These findings are of both clinical and biological significance. From the clinical perspective, they establish that PT2385 has a range of effects on the medical physiology of hypoxia in nonneoplastic tissues, which are manifest at the same doses as antitumor effects reported in mouse xenograft studies (14). Plasma levels of PT2385 achieved using the recommended phase II dose of 800 mg twice daily (15) were similar or higher than those achieved in the mouse study, suggesting that these effects in the mouse are likely relevant to clinical use of the molecule. Recent work has demonstrated effects of a related molecule PT2399 on hypoxia-stimulated erythropoietin production (12, 33). Our work shows marked effects of PT2385 on hypoxic ventilatory control that are as pronounced or more pronounced than the erythropoietic effects in the context of sustained hypoxia. Taken together, these data suggest that PT2385 is likely to have broad effects on physiological and pathophysiological responses that are mediated by HIF-2α. These might include potentially beneficial effects (such as on neovascularization, pulmonary hypertension, or immune activation, ref. 3) or potentially adverse effects, such as the associated anemia that has been reported in clinical trials of PT2385 to date (15, 17). Importantly in this context, the effects on ventilatory control that were observed were highly specific for hypoxia. In unacclimatized mice, we did not observe effects of PT2385 on resting ventilation in normal air or on responses to carbon dioxide. In general, PT2385 was well tolerated. Despite striking reduction in the enhanced ventilatory sensitivity to hypoxia that is observed in mice acclimatized by exposure to 10% oxygen, this hypoxic stress (which is equivalent to an altitude of approximately 5500 m above sea level) was also well tolerated. Consistent with the classical theory on the importance of carbon dioxide in respiratory control, our demonstration of specific effects on responses to hypoxia suggests that PT2385 should not interfere markedly with respiratory control under most circumstances. Nevertheless, the findings suggest that caution will be required in the clinical use of these compounds in some patients, for instance, those with chronic pulmonary diseases and reduced ventilatory sensitivity to carbon dioxide or those at altitude, who may be relying on enhanced sensitivity to hypoxia to maintain stable respiration. Indeed, arterial desaturation was reported in published clinical studies (15, 17), although details of the circumstances surrounding the episodes or the altitudes at which the patients resided were not provided.

The HIF-2α isoform is expressed at unusually high levels in the carotid body (19, 35–37). In keeping with this, several, but not all, genetic studies in the mouse have highlighted the importance of HIF-2α in carotid body responses to hypoxia (19–21, 38). Thus, genetic inactivation of HIF-2α, either generally or specifically in type I cells, has been reported to affect both the enhanced ventilatory sensitivity that is observed with exposure to sustained hypoxia and the proliferative responses of the carotid body that are observed in that setting (19, 20). Inactivation of HIF-2α in carotid body type I cells using Cre-recombinase driven by a TH promoter also leads to severe developmental changes in the carotid body, resulting in a nonfunctional vestigial organ in adult life (21). In contrast, other studies have reported much milder phenotypes, or even enhanced ventilatory sensitivity to hypoxia, in association with heterozygous inactivation of HIF-2α (39, 40). The reasons for these discrepancies among published genetic studies are not known. The current findings provide data, using an orthogonal mode of intervention, corroborating genetic evidence for the importance of HIF-2α in carotid body physiology as well as highlighting the medical implications of its antagonism. Importantly, pharmacological antagonism has also allowed us to assess early time points following HIF-2α inactivation, revealing the surprisingly rapid onset of the effects on ventilatory control.

PT2385 binds to HIF-2α and has been shown to impair its dimerization with HIF-1β, thus preventing the formation of the DNA-binding complex and blocking HIF-2 transcriptional activity (12–14, 31). Most of our findings are consistent with this mode of action. We observed reduction in the expression of *Vegfa* mRNA, which is a HIF-2 target gene in the carotid body (19). Most of the effects of PT2385 were also manifest after a delay that was compatible with an effect on transcriptional output. Thus, it appears likely that transcriptional responses mediated by HIF-2 are responsible, directly or indirectly, for the functional, ultrastructural, and proliferative responses that take place in the carotid body in response to sustained hypoxia and that despite very high levels of HIF-2α in that organ (19, 35–37), these responses to hypoxia are very effectively prevented by PT2385. Nevertheless, some of our findings suggest that the role of HIF-2α in the carotid body may be more complex. First, at higher doses of PT2385, we observed a marked reduction in the baseline hypoxic ventilatory sensitivity in unacclimatized mice, which was manifest within 1 hour of exposure to the compound, perhaps representing an action of HIF-2α (or even another PAS domain–containing protein) on a process more rapid than transcription. Second, we noted that *Epas1^S305M/S305M^* mutant mice showed a hypomorphic phenotype with respect to sensitivity to hypoxia, even in the absence of PT2385 and in spite of unaltered dimerization with HIF-1β. Ventilatory sensitivity in mice is notoriously strain dependent (41, 42). Nevertheless, this mutant allele was carried in the same strain (33) as the mice used in all other studies (19, 20, 25). We were also careful to focus our comparative studies on mice with and without the S305M mutation that were obtained from the same litter. These studies clearly revealed reduced hypoxic ventilatory sensitivity in *Epas1^S305M/S305M^* mice exposed to sustained hypoxia, though responses to carbon dioxide were identical to those of WT animals and their carotid bodies appeared morphologically normal. Proliferative responses of cells within the carotid body were also more variable and somewhat reduced in *Epas1^S305M/S305M^* mice. Interestingly, similar findings were recently reported in *Epas1^S305M/S305M^* mice with respect to erythropoietin production in response to hypoxic stimulation (33). One possibility that has attracted considerable interest and is raised again by the current findings is that the PT2385-binding pocket is the target of an endogenous ligand of HIF-2α that normally contributes to the regulation of transcriptional, or even nontranscriptional, actions of HIF-2α (33, 43). Under this hypothesis, it is possible that the hypomorphic phenotype of *Epas1^S305M/S305M^* mice, with respect to responses to hypoxia, derives from interference with the binding of such a ligand. Resolving whether or not this is the case will require further work. Nevertheless, what is clear from the current work is that molecules targeting HIF-2α in this manner open the way to exploring such possibilities as well as providing an exciting new realm of clinical investigation into the potential utility and risks of their use in nonneoplastic as well as neoplastic conditions that are associated with the activation of HIF-2.

## Methods

### Animals.

Mice were housed in the Functional Genetics Facility of the Wellcome Trust Centre for Human Genetics (University of Oxford) in individually ventilated cages with food and water provided ad libitum and on a 12-hour light/12-hour dark cycle. Male (or, where stated, female) mice aged approximately 8 weeks were used in all experiments; all genotypic comparisons were with littermate and sex-matched controls. *Epas1^S305M/S305M^* mice (as described in ref. 33), *Phd2^+/–^* mice (as described in ref. 25), and WT mice were on a C57BL/6 genetic background.

### Sustained hypoxic exposure of mice and drug administration.

For mouse experiments, PT2385 ([S]-3-[(2,2-difluoro-1-hydroxy-7-[methylsulfonyl]-2,3-dihydro-1 H-inden-4-yl)oxy]-5-fluorobenzonitrile) (MedChemExpress) was suspended in 5 mg/mL methyl cellulose (Sigma) with 0.5% Tween 80 (Sigma) to a final concentration of 0.75 mg/mL or 2.5 mg/mL, as described previously (14). All mice were treated twice daily by oral gavage with 3 or 10 mg/kg PT2385 (4 mL/kg) or an equivalent volume of vehicle as control. Mice were given 50 mg/kg BrdU (Sigma) dissolved in aqueous phosphate buffer pH 7.0 (Fisher Scientific) via i.p. injection immediately before exposure to hypoxia or normoxia, followed by supplementation of drinking water with BrdU (1 mg/mL and 1% sucrose) for the 7-day experimental period. For sustained exposure to hypoxia, mice were placed into a normobaric altitude chamber and acclimatized to decreasing oxygen levels over 24 hours and then maintained at 10% oxygen for 7 days with controlled temperature, humidity, and carbon dioxide levels and free access to food and water (25). Mice were briefly removed from the normobaric altitude chamber in order to perform plethysmography measurements (30 minutes in normal air, then 30 minutes in unsealed plethysmographs followed by 3 cycles of the following: 5 minutes medical air/5 minutes acute gas challenge/5 minutes medical air with 15 minutes in unsealed plethysmographs between the cycles) and/or oral treatment with PT2385 or vehicle. Normoxic littermate controls were maintained under normal housing conditions. All mice were weighed at the start of the procedure and monitored daily for general condition and changes in body weight. No significant differences in body weight were noted with PT2385 treatment and/or in *Epas1^S305M/S305M^* mice.

### Plethysmography.

Tidal volume and respiratory rate were measured in awake, unrestrained mice using whole-body plethysmographs (600 mL; catalog PLY4211; Buxco) (25). Minute ventilation was calculated from tidal volume and respiratory rate. Ventilatory parameters were derived using FinePointe software (Buxco) and normalized to body weight as measured immediately before plethysmography. Premixed gas was delivered to each chamber as described previously (25). The acute gas challenge consisted of 10% oxygen, balance nitrogen; 10% oxygen, 3% carbon dioxide, balance nitrogen; or 21% oxygen, 3% carbon dioxide, balance nitrogen. The AVR to each stimulus was defined as the difference between minute ventilation during the 1 minute before the onset of acute gas challenge and the first 1 minute of stable acute gas exposure (following a 30-second delay period after switching the gas mixtures).

For unacclimatized measurements, mice were treated with the first dose of PT2385 (or vehicle) and ventilation was measured by plethysmography 1 and 5 hours following this dose, after which the second dose of PT2385 (or vehicle) was administered. The following day, ventilation was measured 24 hours following the first dose.

For measurements of ventilatory sensitivity in animals exposed to sustained hypoxia, mice were given PT2385 (or vehicle) twice daily for 24 hours, then placed into the normobaric altitude chamber and twice daily PT2385 (or vehicle) treatment continued. Plethysmography measurements were made following 2, 5, and 7 days of sustained hypoxia in the morning, before the first daily dose of PT2385.

### Blood measurements.

Blood was obtained from the inferior vena cava of mice using heparinized capillary tubes after terminal anesthesia and hematocrits measured using a hematocrit centrifuge (catalog C-MH30; Unico).

### Tissue preparation.

Mice were killed by overdose of isoflurane (Piramal Critical Care) (in the altitude chamber for the hypoxic mice) and exsanguination. This was followed by dissection of tissues, which were then immersion-fixed in 4% paraformaldehyde/PBS (Sigma) overnight. Tissues were then transferred to 70% ethanol and progressively dehydrated in an ascending ethanol series before paraffin embedding. Paraffin-embedded tissues were sectioned to 4 μm using an RM135 microtome (Leica Biosystems).

### Immunohistochemistry and in situ hybridization.

Paraffin-embedded sections were immunostained with anti-TH (dilution 1:5000; NB300-109; Novus Biologicals) or with an anti-BrdU antibody (dilution 1:10; catalog 551321; BD Biosciences), as described previously (25). *Vegfa* and *Hif-2α* mRNA transcripts were detected via in situ hybridization on sections from paraffin-embedded tissues using the manual RNAscope 2.5 HD BROWN assay (Advanced Cell Diagnostics). Samples were imaged using a DM 1000 LED microscope (Leica Biosystems). Stereological estimation of cell number, cell density, and carotid body volume was performed using ImageJ (NIH) on every fourth paraffin section (25). In situ hybridization probe signal was quantified using trainable Weka segmentation in FIJI ImageJ software (NIH), according to the manufacturer’s instructions.

### EM.

Tissue samples for EM were harvested immediately after terminal anesthesia and immersion-fixed in 4% formaldehyde and 2.5% glutaraldehyde in 0.1 M phosphate buffer pH 7.4 for 1 hour at room temperature. Tissue was post-fixed in 2% aqueous osmium tetroxide for 1 hour at 4°C. Tissue was rinsed in water, stained with 2% aqueous uranyl acetate overnight at 4°C, dehydrated in an ascending ethanol series, infiltrated with epon resin mixed with propylene oxide, and embedded in 100% epon resin. To determine the location of the carotid body, the polymerized sample was imaged using microCT (Zeiss Xradia 510 Versa) with a Zeiss filter LE2 and a ×4 objective lens. To confirm the presence of the carotid body by light microscopy, semithin sections were produced and stained using toluidine blue, as described previously (44). Ultrathin sections were produced and stained, as described previously (45). EM imaging was performed using a Tecnai G2 Spirit BioTWIN transmission EM (Thermo Fisher Scientific) equipped with a OneView CMOS camera (Gatan).

### Quantitative real-time PCR.

Carotid artery bifurcations were dissected from 5 littermate pairs of WT and *Epas1^S305M/S305M^* mice maintained in 10% oxygen for 7 days and transferred into ice-cold PBS. Carotid bodies were subdissected and stored in RNA*later* (QIAGEN) on ice. Carotid bodies from all animals per genotype were then pooled, and RNA was isolated and cDNA prepared as described previously (35). *Hif-2α* mRNA was quantified in triplicates in a duplex quantitative real-time PCR reaction using TaqMan Fast Advanced Master Mix and *Hif-2α* (*Epas1*) FAM and *Actb* VIC TaqMan Gene Expression Assays (Mm00438712_m1 and Mm01205647_g1, Thermo Fisher Scientific). The reaction was carried out in the StepOnePlus Real-Time PCR System (Applied Biosystems). ΔC_T_ was defined as the difference between the *Epas1* C_T_ and the *Actb* C_T_. –ΔΔC_T_ values were calculated for each replicate as follows: -(*Epas1^S305M/S305M^* ΔC_T_ – WT ΔC_T_) (46). Fold change in the *Epas1* mRNA expression in each genotype group was expressed as 2^–ΔΔCT^.

### Plasmid construction and site-directed mutagenesis.

Mouse HIF-2α PAS-B domain (GenBank accession AAH57870, residues 241–361) was cloned into pSJ2 vector, as previously described (31). Mutation of S305M was introduced using the QuikChange II XL Site-Directed Mutagenesis Kit (Agilent).

### Protein expression and purification.

WT mouse HIF-2α PAS-B domain and the S305M mutation were both expressed and purified, as previously described (31). HIF-2α/HIF-1β heterodimers (WT and with the S305M mutation in the HIF-2α subunit) consisting of bHLH-PAS-A-PAS-B domains were expressed and purified using the coexpression constructs and procedures previously published (31).

### ITC.

PT2385 (Abcam) was initially dissolved in DMSO and then diluted 1:1000 in buffer with 20 mM Tris pH 8.0 and 150 mM NaCl. Temperatures were measured on a Nano ITC system (TA Instruments), with HIF-2α PAS-B domains in 20 mM Tris pH 8.0, 150 mM NaCl, and 0.1% DMSO at 20°C. 30 aliquots of 7.98 μL of 200 μM HIF-2α PAS-B domain, or the S305M mutation, were injected into 40 μM PT2385. Stoichiometry, binding constant, and change in enthalpy of interaction were calculated using the ITCRun and NanoAnalyze software (TA Instruments).

### Protein thermal shift.

Experiments were carried out on a StepOnePlus Real-Time PCR System (Applied Biosystems) using 2 μM of each protein construct in buffer containing 20 mM Tris pH 8.0 and 150 mM NaCl, as previously described (31). Data were analyzed using the DMAN, version 5.2 (47), to calculate ΔT_m_.

### Statistics.

Data are shown as mean ± SEM, unless stated otherwise. Details of the statistical tests used are included in the respective figure legends. *P* < 0.05 was considered statistically significant throughout and *P* values corrected for multiple comparisons as indicated. All statistical analyses were performed using GraphPad Prism, version 8.2.0.

### Study approval.

The animal studies were performed under the Home Office animal work project license (PPL P38BE32DE) in accordance with protocols approved by both the Ethics Committee of the University of Oxford and the Home Office Animals (Scientific Procedures) Act 1986, United Kingdom.

## Author contributions

Experiments were designed by TB and PJR. Data were predominantly collected and analyzed by XC, MPB, JWF, MZ, ALB, JDCCL, LE, ILAA, RKB, LMC, FR, TB, and PJR with contributions from other authors. The manuscript was written by TB and PJR with input from XC, MPB, JWF, CWP, KJB, PAR, EJH, and FR and reviewed by all authors. Figures were prepared and statistical analyses were performed by MPB with input from other authors. Order of co–first authors is based on the length of time spent on the project. Co–senior authors TB and PJR conceived the study and managed the project throughout.

## Supplementary Material

Supplemental data

## Figures and Tables

**Table 1:**
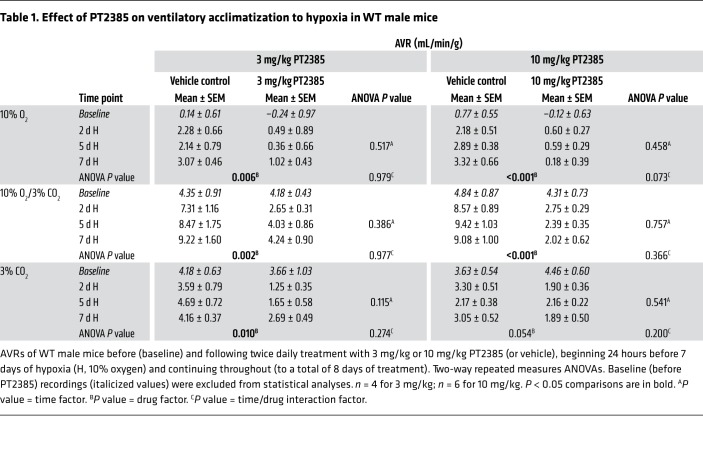
Effect of PT2385 on ventilatory acclimatization to hypoxia in WT male mice

**Table 2:**
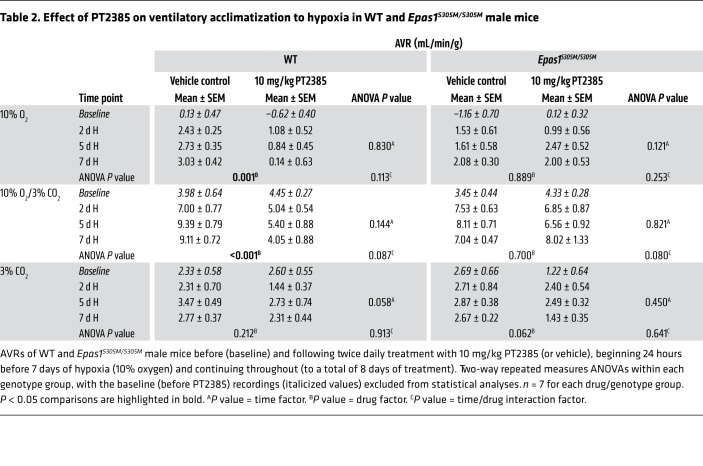
Effect of PT2385 on ventilatory acclimatization to hypoxia in WT and *Epas1^S305M/S305M^* male mice

## Version 1 01/30/2020

In-Press Preview

## Version 2 03/23/2020

Electronic publication

## Version 3 05/01/2020

Print issue publication
