# Supplementary material for: Marked and rapid effects of pharmacological HIF-2α antagonism on hypoxic ventilatory control
Source: J Clin Invest. 2020 Mar 23;130(5):2237–51. doi: 10.1172/JCI133194 (PMC7190921; doi:10.1172/JCI133194)
Supplement: Supplemental data [file jci-130-133194-s037.pdf]

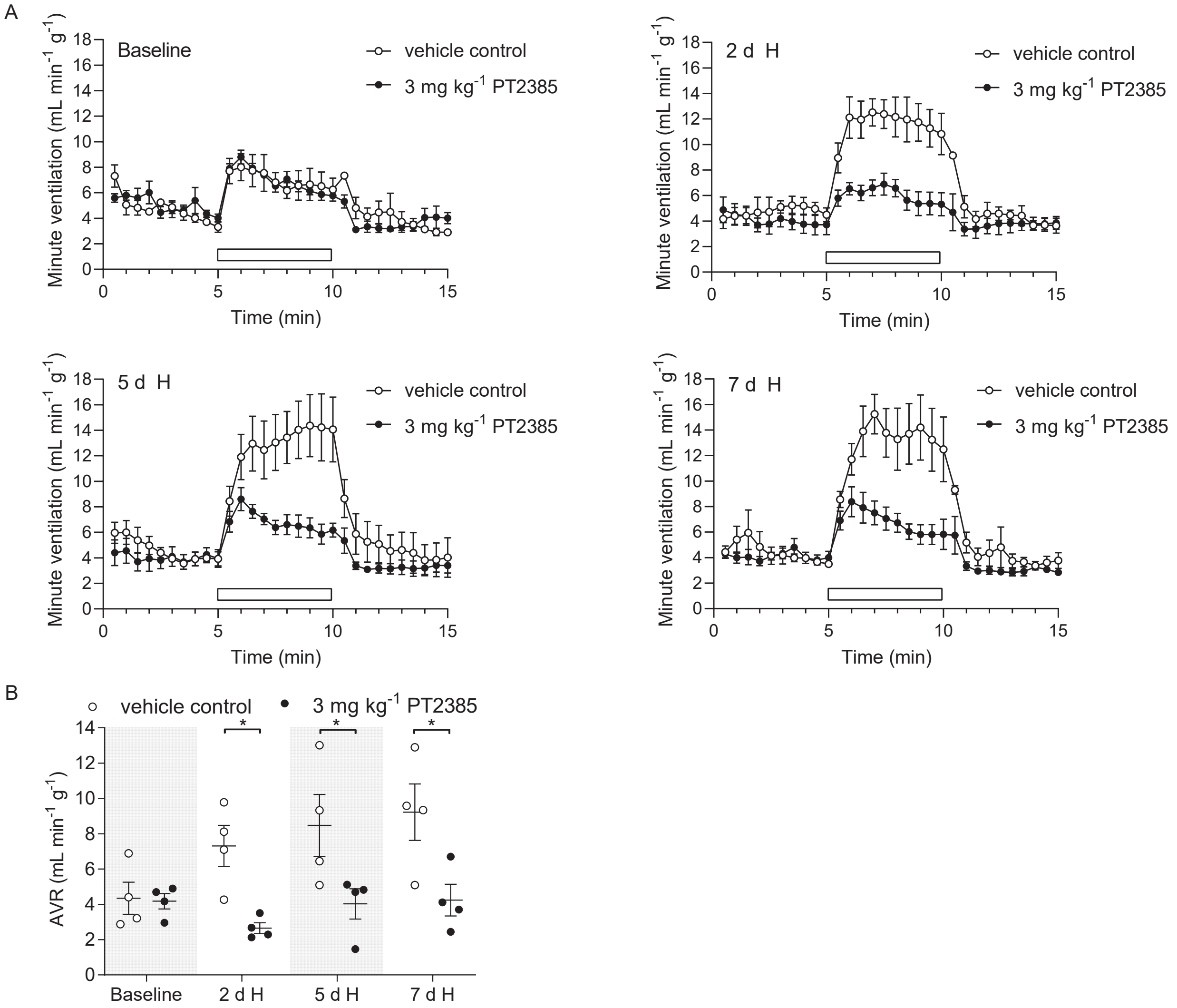

Figure S1. Loss of ventilatory acclimatisation to hypoxia in male mice treated with 3 mg kg^{-1} PT2385.

(**A**) Graphs show changes in minute ventilation in response to an acute (5 min) challenge with 10 % O_2/ 3 % CO_2 (open bars) in mice before (Baseline) and following twice daily treatment with 3 mg kg^{-1} PT2385 (or vehicle), beginning 24 h before 7 d exposure to hypoxia (H, 10 % oxygen) and continuing throughout (to a total of 8 days treatment)(n = 4). (**B**) Graph shows acute ventilatory responses (AVRs) to challenges with 10 % O_2/ 3 % CO_2, quantified from the minute ventilation data shown in (**A**). Data were analysed by a two-way repeated measures ANOVA with Baseline recordings removed from statistical analysis (P values shown in Table 1); followed by Holm-Sidak's multiple comparisons two-tailed test for which the significance is reported in the graph, * $P < 0.05$.

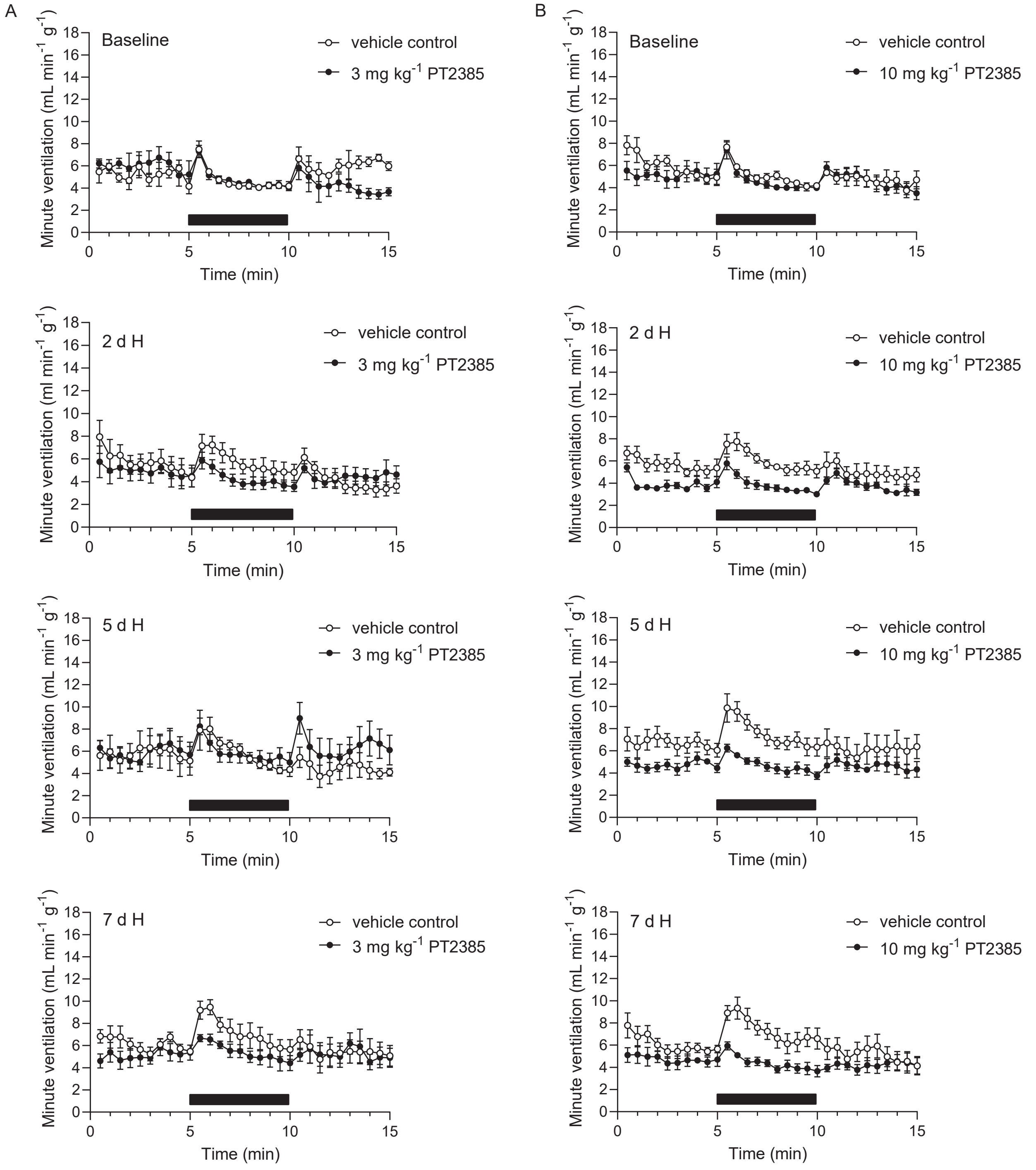

Figure S2. Effects of PT2385 on enhanced ventilatory sensitivity to hypoxia in male mice as measured by acute challenge with 10 % oxygen.

Graphs show changes in minute ventilation in response to an acute (5 min) challenge with 10 % O_2 (black bars) in mice before (Baseline) and following twice daily treatment with (**A**) 3 or (**B**) 10 mg kg^{-1} PT2385 (or vehicle), beginning 24 h before 7 d exposure to hypoxia (H, 10 % oxygen) and continuing throughout (to a total of 8 days treatment)(n = 4, 3 mg kg^{-1}; n = 6, 10 mg kg^{-1}).

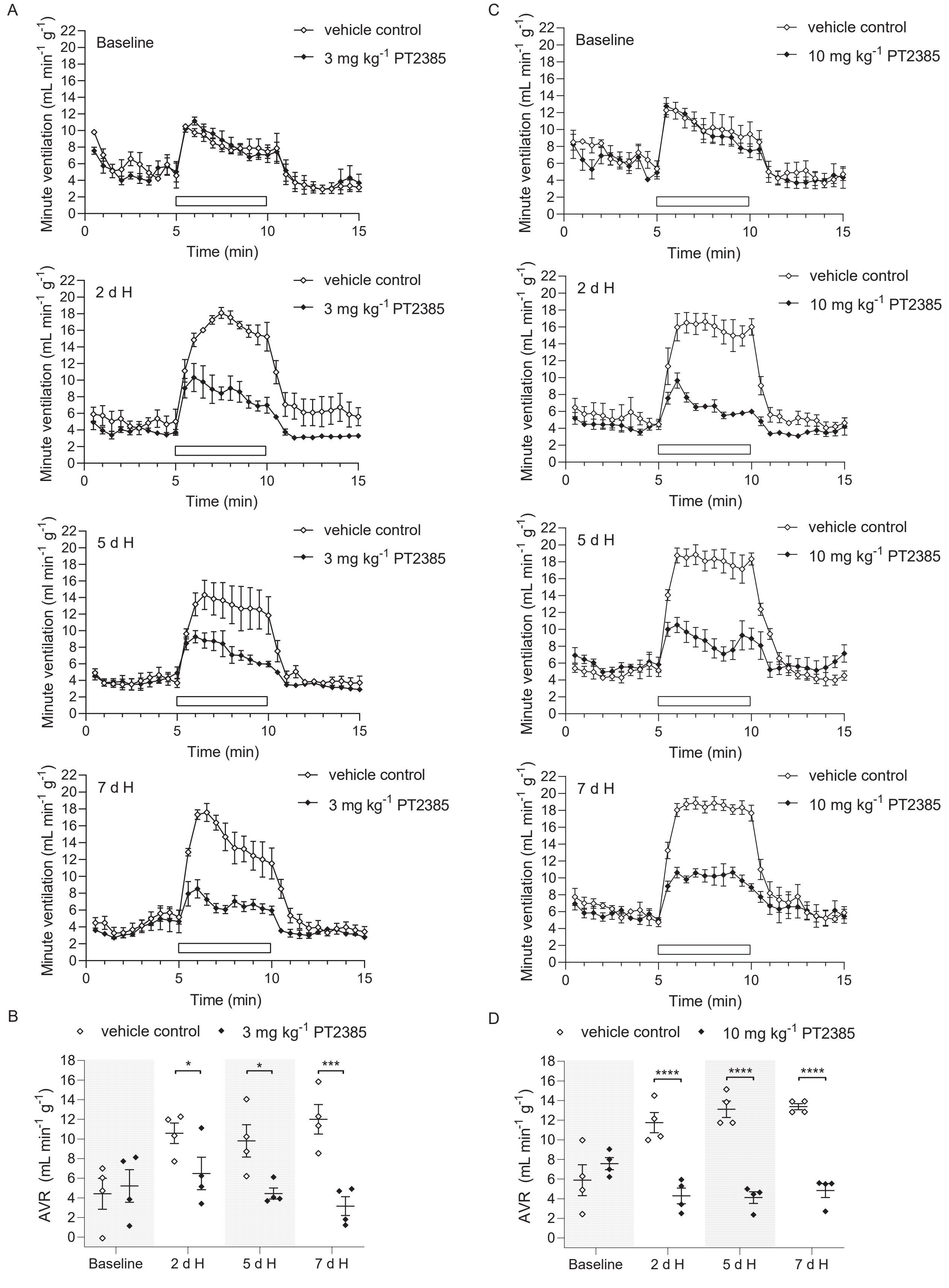

Figure S3. Loss of ventilatory acclimatisation to hypoxia in female mice treated with PT2385.

Graphs show changes in minute ventilation in response to an acute (5 min) challenge with 10 % O_2/ 3 % CO_2 (open bars) in mice before (Baseline) and following twice daily treatment with (**A**) 3 or (**C**) 10 mg kg^{-1} PT2385 (or vehicle), beginning 24 h before 7 d exposure to hypoxia (H, 10 % oxygen) and continuing throughout (to a total of 8 d treatment)(n = 4). (**B, D**) Graphs show acute ventilatory responses (AVRs) to challenges with 10 % O_2/ 3 % CO_2, quantified from the minute ventilation data shown in (**A, C**). Data analysed by a two-way ANOVA matched by the time factor and with Baseline recordings removed from statistical analysis (P values shown in Table S1); followed by Holm-Sidak's multiple comparisons two-tailed tests for which the significance is reported in the graph, * $P < 0.05$, *** $P < 0.001$, **** $P < 0.0001$.

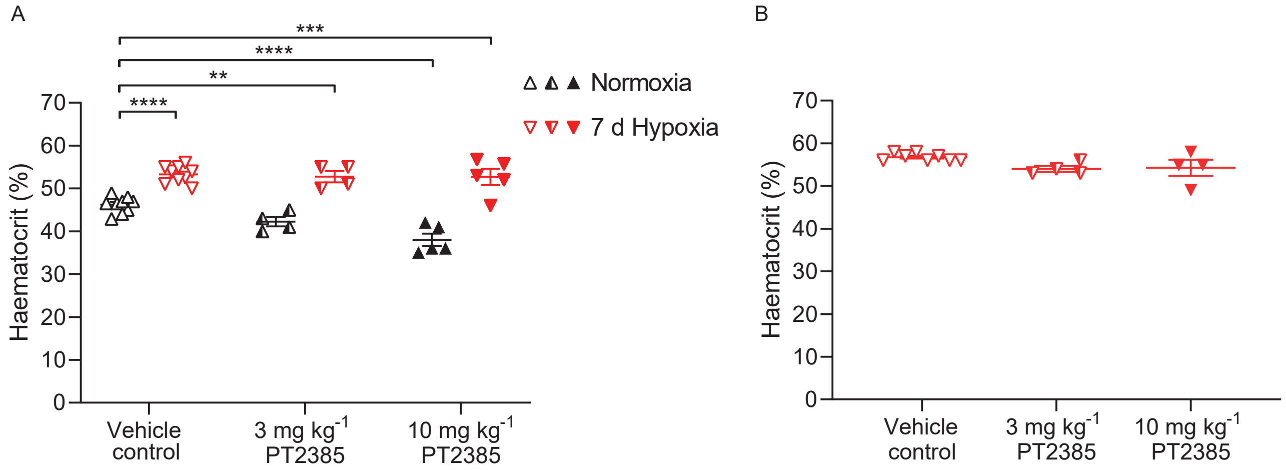

Figure S4. Changes in haematocrit in wild-type mice treated with 3 or 10 mg kg^{-1} PT2385 and maintained in normoxia or exposed to sustained hypoxia.

Graphs show % haematocrit in the blood of wild-type (**A**) male and (**B**) female mice treated with 3 or 10 mg kg^{-1} PT2385 beginning 24 h before, and continuing throughout, 7 d exposure to hypoxia (10 % oxygen), or normoxia (to a total of 8 d treatment). Data were analysed by a two-way (males) or one-way (females) ANOVA; followed by Holm-Sidak's multiple comparisons two-tailed tests, ** $P < 0.01$, *** $P < 0.001$, **** $P < 0.0001$.

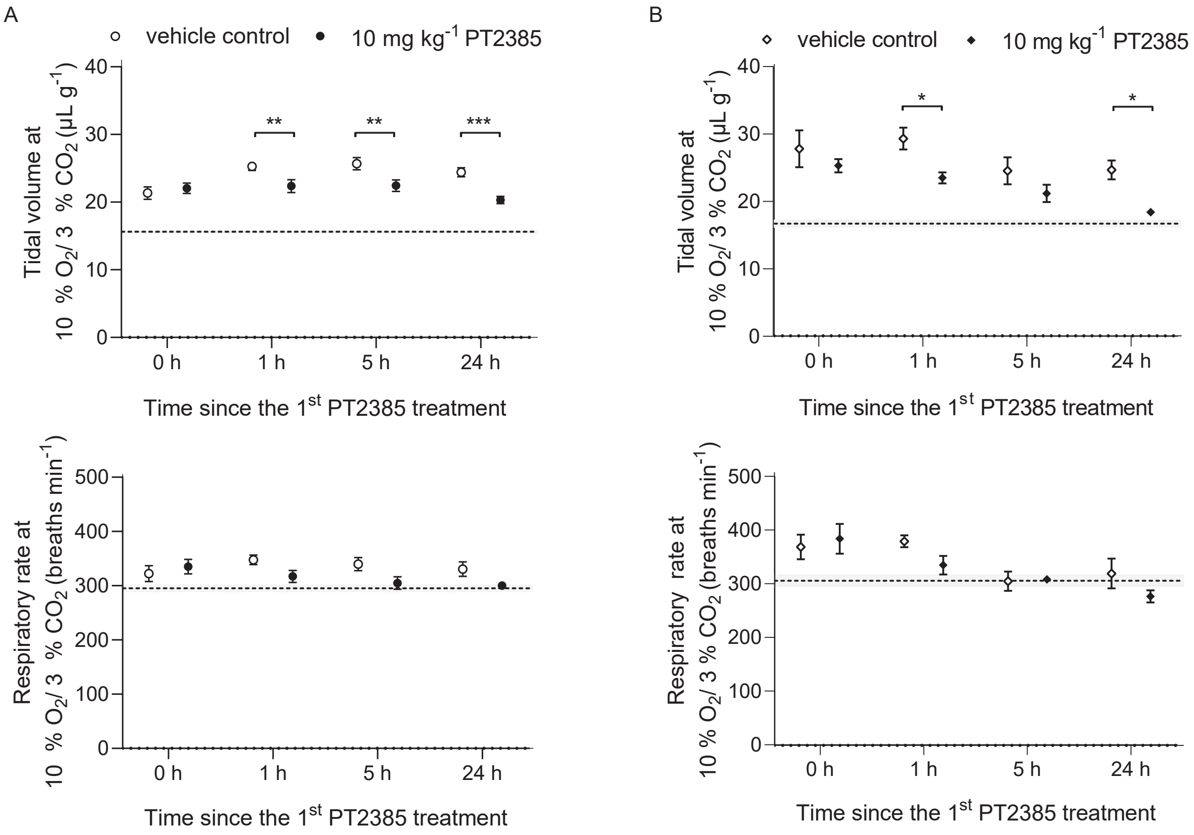

Figure S5. Changes in tidal volume and respiratory rate in unacclimatised PT2385-treated mice.

Graphs show average tidal volume (top panels) and respiratory rate (bottom panels) during 5 min challenges with 10 % O_2/ 3 % CO_2. Measurements were made before (0 h) and 1, 5 and 24 h after the first 10 mg kg^{-1} PT2385 (or vehicle) dose in (**A**) male (n = 15) and (**B**) female mice (n = 4); second dose of PT2385 was given immediately after the 5 h measurements. Data were analysed by Holm-Sidak's multiple comparisons two-tailed tests, * $P < 0.05$, ** $P < 0.01$, *** $P < 0.001$. Dotted line shows average resting tidal volume or respiratory rate in air across all time-points and treatment groups, ± SEM (shaded area).

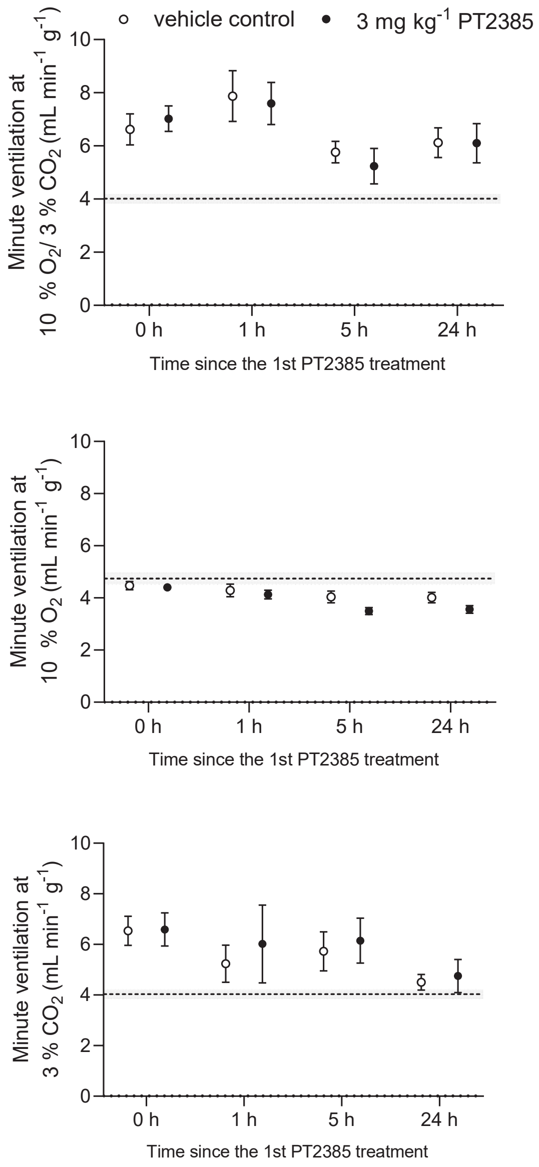

Figure S6. Effects of 3 mg kg^{-1} PT2385 on ventilatory sensitivity in unacclimatised male mice.

Graphs show average minute ventilation during 5 min challenges with 10 % O_2/ 3 % CO_2 (upper panel), 10 % O_2 (middle panel) or 3 % CO_2 (lower panel). Measurements were made before (0 h) and 1, 5 and 24 h after the first 3 mg kg^{-1} PT2385 (or vehicle) dose (n = 8); second dose of PT2385 was given immediately after the 5 h measurements. Data were analysed by Holm-Sidak's multiple comparisons two-tailed tests with no significant effects detected. Dotted lines show the average resting minute ventilation in air prior to the acute gas challenge, across all time-points and treatment groups depicted in that graph, ± SEM (shaded area).

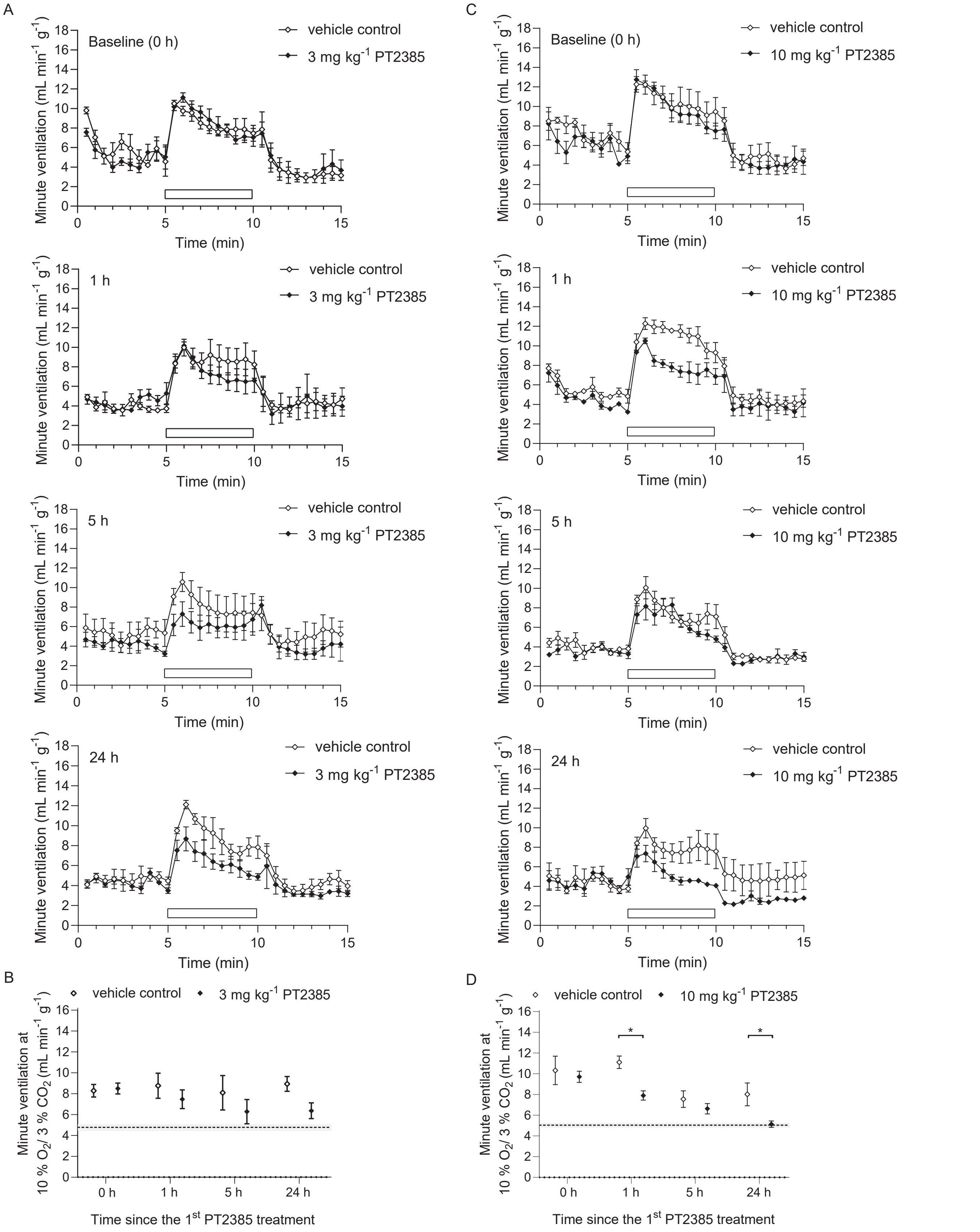

Figure S7. Rapid effects of PT2385 on acute ventilatory responses to hypoxia in wild-type female mice.

Graphs show changes in minute ventilation in response to an acute (5 min) challenge with 10 % O_2/ 3 % CO_2 (open bars). Measurements were made before (Baseline, 0 h) and 1, 5 and 24 h after the first (**A**) 3 or (**C**) 10 mg kg^{-1} PT2385 (or vehicle) dose (*n* = 4); second dose of PT2385 was given immediately after the 5 h measurements. (**B, D**) Graphs show average minute ventilation during 5 min challenges with 10 % O_2/ 3 % CO_2 (calculated from data in **A, C**). Data were analysed by Holm-Sidak's multiple comparisons two-tailed tests, * $P < 0.05$. Dotted lines show the average resting minute ventilation in air prior to the acute gas challenge, across all time-points and treatment groups depicted in that graph, ± SEM (shaded area).

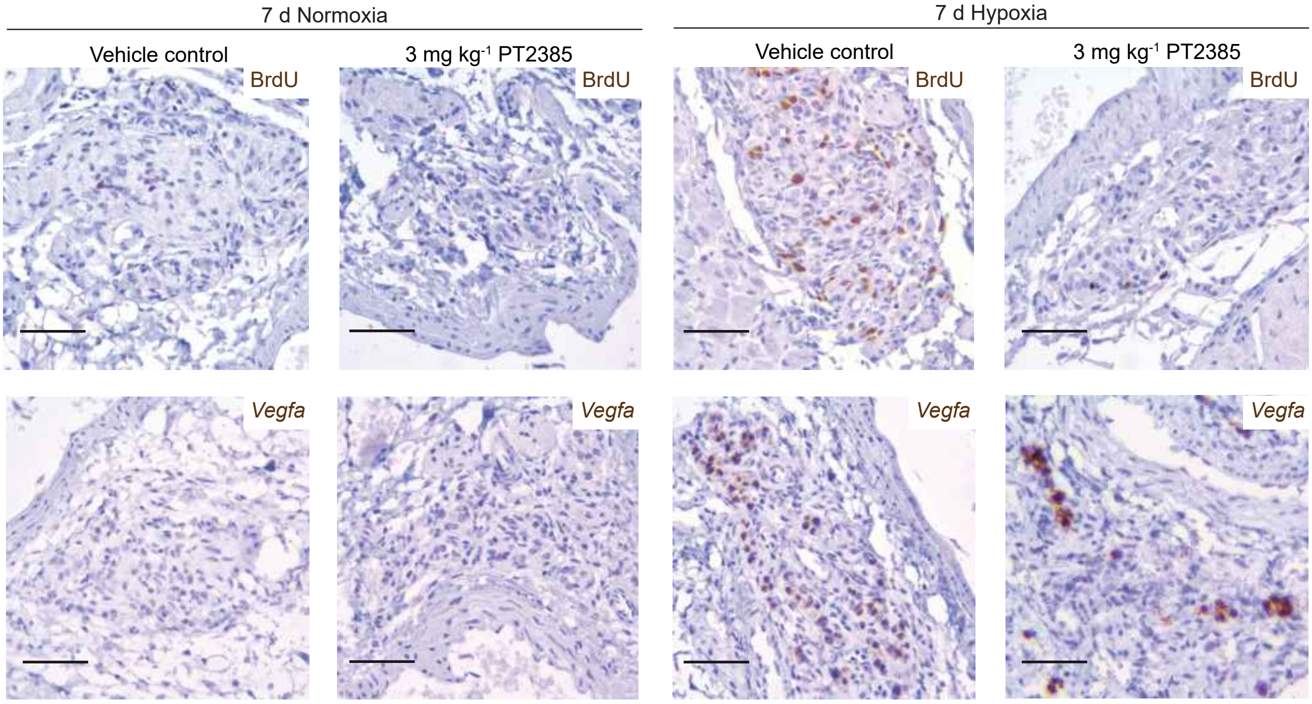

Figure S8. Responses to hypoxia in carotid bodies of male mice treated with 3 mg kg^{-1} PT2385.

Representative images of immunostaining for BrdU and in situ hybridisation for *Vegfa* mRNA in carotid bodies of mice treated twice daily with 3 mg kg^{-1} PT2385 (or vehicle) beginning 24 h before, and continuing throughout, 7 d exposure to hypoxia (10 % oxygen) or normoxia (to a total of 8 d treatment). Number of BrdU+ cells/ CB area and *Vegfa* mRNA+ CB area quantified in Figure 4A. Scale bars represent 50 µm.

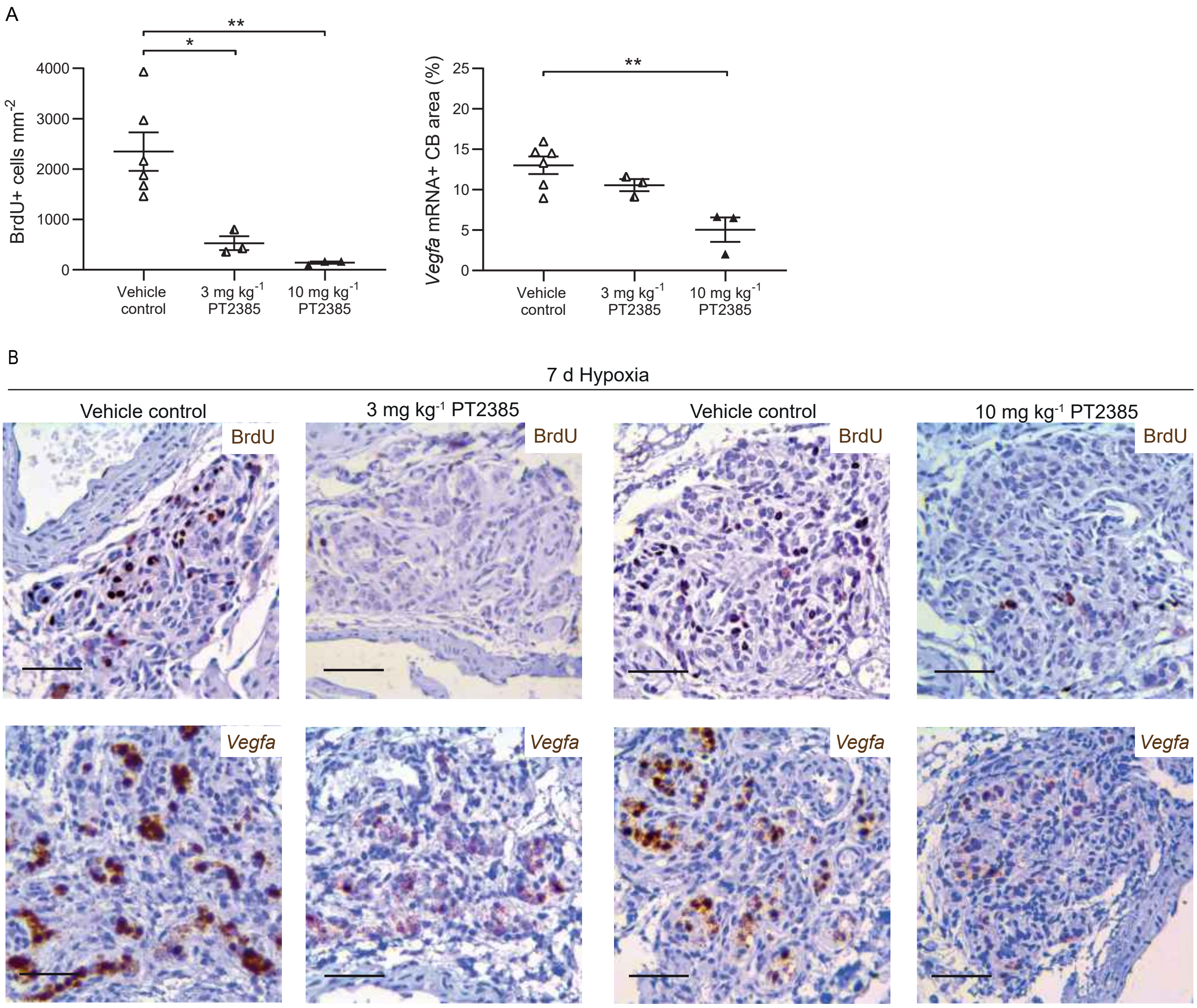

Figure S9. Effects of PT2385 on cellular responses to hypoxia in the carotid bodies of female mice.

(**A**) Morphometry and (**B**) representative images of carotid bodies (CBs) from wild-type female mice exposed to 7 d hypoxia (10 % oxygen) and treated twice daily with 3 or 10 mg kg^{-1} PT2385 (or vehicle) for 8 d. (**A**) Quantification of BrdU+ cells per mm^2 (left panel) and *Vegfa* mRNA+ CB area of the CB (right panel). Data were analysed by one-way ANOVAs followed by Holm-Sidak's multiple comparisons two-tailed tests, for which significance is reported in the graphs, * $P < 0.05$, ** $P < 0.01$. (**B**) Immunostaining for BrdU and *Vegfa* mRNA in situ hybridisation. Scale bars represent 50 µm.

Table S1. Effect of PT2385 on ventilatory acclimatisation to hypoxia in wild-type female mice.

		AVR (mL min^{-1} kg^{-1})									
		3 mg kg^{-1} PT2385					10 mg kg^{-1} PT2385				
		Vehicle control		3 mg kg^{-1} PT2385		ANOVA	Vehicle control		10 mg kg^{-1} PT2385		ANOVA
	Time-point	Mean	SEM	Mean	SEM	*P* value	Mean	SEM	Mean	SEM	*P* value
10% O_2	Baseline	-0.35	± 0.31	0.34	± 0.99		-0.52	± 1.00	-0.25	± 0.77	
	2 d H	1.85	± 0.60	0.72	± 0.38		1.34	± 0.53	-0.10	± 0.66	
	5 d H	2.74	± 0.91	0.19	± 0.65	*0.976*	3.33	± 1.35	-1.90	± 0.34	*0.370*
	7 d H	3.51	± 1.41	-1.01	± 1.21		3.88	± 0.74	-0.74	± 0.39	
	ANOVA *P* value	***0.002***				*0.301*	***0.001***				***0.043***
10% O_2 / 3% CO_2	Baseline	4.42	± 1.58	5.22	± 1.66		5.89	± 1.58	7.57	± 0.61	
	2 d H	10.58	± 1.04	6.49	± 1.65		11.75	± 1.03	4.29	± 0.80	
	5 d H	9.81	± 1.64	4.44	± 0.56	*0.564*	13.12	± 0.84	4.12	± 0.59	*0.342*
	7 d H	12.01	± 1.51	3.16	± 0.96		13.38	± 0.31	4.84	± 0.71	
	ANOVA *P* value	***0.001***				*0.212*	***<0.001***				*0.553*
3% CO_2	Baseline	6.97	± 1.04	6.83	± 1.66		5.29	± 0.62	5.79	± 0.88	
	2 d H	2.92	± 0.30	2.41	± 0.24		4.63	± 0.79	2.94	± 0.87	
	5 d H	5.01	± 0.80	2.47	± 0.68	*0.125*	6.63	± 0.89	3.95	± 0.24	*0.287*
	7 d H	4.80	± 0.87	2.65	± 0.56		4.98	± 0.71	3.71	± 1.40	
	ANOVA *P* value	***0.030***				*0.193*	***0.032***				*0.736*

Acute ventilatory responses (AVRs) of wild-type female mice before (Baseline) and following twice daily treatment with 3 mg kg^{-1} or 10 mg kg^{-1} PT2385 (or vehicle), beginning 24 h before 7 d hypoxia (H, 10 % oxygen) and continuing throughout (to a total of 8 d treatment). Two-way repeated measures ANOVAs (right hand column *P* value = time factor; bottom row *P* value = drug factor; right column, bottom row *P* value = time/ drug interaction factor), matched by the time factor, with the Baseline (prior to PT2385) recordings excluded from statistical analysis; $n = 4$. $P < 0.05$ comparisons are highlighted in bold.
